# Fabrication of Mn_3_O_4_-CeO_2_-rGO as Nanocatalyst for Electro-Oxidation of Methanol

**DOI:** 10.3390/nano12071187

**Published:** 2022-04-02

**Authors:** Mohammad Bagher Askari, Seyed Mohammad Rozati, Antonio Di Bartolomeo

**Affiliations:** 1 Department of Physics, Faculty of Science, University of Guilan, Rasht P.O. Box 41335-1914, Iran; mbaskari@phd.guilan.ac.ir; 2 Department of Physics “E. R. Caianiello” and Interdepartmental Center NANOMATES, University of Salerno, 84084 Fisciano, SA, Italy

**Keywords:** Mn_3_O_4_-CeO_2_-rGO, nanocatalyst, methanol oxidation, cyclic voltammetry

## Abstract

Recently, the use of metal oxides as inexpensive and efficient catalysts has been considered by researchers. In this work, we introduce a new nanocatalyst including a mixed metal oxide, consisting of manganese oxide, cerium oxide, and reduced graphene oxide (Mn_3_O_4_-CeO_2_-rGO) by the hydrothermal method. The synthesized nanocatalyst was evaluated for the methanol oxidation reaction. The synergetic effect of metal oxides on the surface of rGO was investigated. Mn_3_O_4_-CeO_2_-rGO showed an oxidation current density of 17.7 mA/cm^2^ in overpotential of 0.51 V and 91% stability after 500 consecutive rounds of cyclic voltammetry. According to these results, the synthesized nanocatalyst can be an attractive and efficient option in the methanol oxidation reaction process.

## 1. Introduction

The increasing energy need and the scarcity of fossil fuels have pushed countries towards the use of clean and natural fuel sources and alternative energy resources [1,2]. The excessive consumption of fossil fuels has caused global warming and an increase in greenhouse gases, thus posing very serious environmental risks to human health and the planet [3,4,5].

The use of clean, available, natural, and cost-effective fuels is one of the best suggestions to overcome these environmental crises [6]. In recent years, the use of renewable energy sources such as sunlight and wind energy may have become more widely available and accessible than other renewable sources [7]; furthermore, various sciences have introduced attractive and portable devices, which open new avenues in the field of energy storage and conversion. The use of various types of electrochemical batteries, supercapacitors, and fuel cells are among these devices [8,9].

Fuel cells convert directly chemical energy to electrical energy and have high efficiency [10], while supercapacitors and electrochemical batteries are used to store energy [11]. One of the most important challenges for researchers is the introduction of inexpensive materials for use in electrodes of supercapacitors and electrochemical batteries as well as fuel cells.

The introduction of high-efficiency and cost-effective materials in the field of energy storage has almost been achieved, and various studies have confirmed this claim [12,13,14]. However, in the field of catalysts, especially catalysts in alcohol fuel cells, the introduction of high-efficiency and low-cost catalysts has not been fully possible so far. The materials based on platinum, palladium, ruthenium, and other rare and precious metals are the best catalysts in methanol oxidation [15], and finding a catalyst that can compete with these materials requires more effort and research. It should be noted that many cost-effective catalysts have been introduced in the field of methanol oxidation, although small amounts of precious metals have been often used in the catalyst structure. Moreover, composing and hybridizing the Pt, Pd, Ru, etc., with materials such as carbon, metal oxides, metal sulfides, and conductive polymers have been reported in many works [16,17,18,19,20].

For the introduction of cheap platinum-free catalysts, metal oxides have recently received much attention [21]. Although these materials suffer from poor electrical conductivity and never have the electrocatalytic activity that can compete with commercial catalysts for the oxidation of methanol, they could have relatively good catalytic activity in the oxidation of methanol by some modifications [22,23,24]. Among the transition metal oxides, manganese oxides such as MnO_2_, Mn_2_O_3_, and Mn_3_O_4_ have received significant attention in different applications due to their cost-effective, eco-friendly, and electrochemical properties [25]. For example, the development of metal oxides including Mn_3_O_4_ nanoparticles [25], hollow-like Mn_3_O_4_ nanostructures on graphene matrix [26], Fe-doped Mn_3_O_4_ nanoboxes [27], and Ce-doped Mn_3_O_4_ [28] for methanol oxidation, oxygen reduction, and oxygen evolution reactions has been considered by researchers.

Cerium oxide has also recently received much attention for catalyst and sensing applications. The placement of CeO_2_ in the structure of catalysts has always improved their electrochemical properties. Applications of this material in the form of multi-component composites that have studied the synergetic effect of CeO_2_ with other materials in the catalyst include the use of NiCoO_2_@ CeO_2_ nanoboxes in ultrasensitive electrochemical immunosensing based on oxygen evolution reaction [29] or the application of CeO_2_@ CoP as a supercapacitor electrode [30]. Other studies have been reported including the use of CeO_2_-ZnO in low-temperature solid oxide fuel cells [31] and extensive use of CeO_2_-based material in electrochemical water splitting [32]. In addition, CeO_2_ has been reported as a filler in proton-exchange membranes, for which one can refer to the research of Vinothkannan et al. [33]. CeO_2_ is one of the metal oxides used in the catalyst materials for the methanol oxidation process, for example, the application of Pt/CeO_2_ [34], Pd-CeO_2_ [35], and Au@ CeO_2_ [36] in the MOR process. In these cases, CeO_2_ has been used as a catalyst along with precious and rare metals. However, the use of this material along with inexpensive materials has also been studied as a catalyst for methanol oxidation, including the use of CeO_2_-decorated rGO [37] and CeO_2_/NiO hollow spheres [38]. According to the literature, CeO_2_ has always been considered in most electrochemical fields, including various types of electrochemical sensors or energy production and storage devices.

Moreover, we investigated NiCo_2_O_4_ nanorods/reduced graphene oxide (rGO) [39], MoS_2_/Ni_3_S_2_/rGO [40], NiO-Co_3_O_4_-rGO [41], and MgCo_2_O_4_/rGO [42] as catalysts for methanol oxidation in previous works, and our results show that the synergetic effect between metal oxides/sulfides and reduced graphene oxide improves the catalytic activity of the catalyst. These catalysts were synthesized by the hydrothermal method. In this method, time and temperature are two very important factors that by controlling these parameters, a synthesis with very good efficiency and desired morphology can be achieved. In many studies, by changing these two parameters, nanomaterials with different sizes, morphology, and porosity have been obtained [43,44].

In this work, we study a hybrid consisting of a mixed metal oxide (Mn_3_O_4_ and CeO_2_) and reduced graphene oxide (rGO) as a catalyst for use in the methanol oxidation process. We investigate the synergetic effect of rGO with Mn_3_O_4_-CeO_2_ on the electrochemical properties of catalyst in the methanol oxidation process. RGO was added to increase the active surface area and the conductivity of the catalyst. Electrochemical tests indicate the synergetic effect of composite components on the oxidation of methanol. It seems that this catalyst, due to its good capability in the methanol oxidation process, can be evaluated as a stable and relatively efficient catalyst in the anode structure of methanol fuel cells.

## 2. Materials and Methods

### 2.1. Materials and Instruments

Cerium nitrate (Ce(NO_3_)_3_ · 6H_2_O), potassium permanganate (KMnO_4_), and urea were used as precursors for the synthesis of catalysts and used with high purity. Precursors were purchased from Merck company (Merck, Darmstadt, Germany). X-ray diffraction analysis was performed by a Thermo Scientific device (Thermo Fisher Scientific, ARL Equinox 3000, Waltham, MA, USA), and scanning electron microscopy (SEM) was performed by FEI Quanta 200 FEG-Netherlands. Electrochemical tests were conducted by potentiostat–galvanostat AUTOLAB PGSTAT 302 N (“Metrohm Autolab bv”, Utrecht, The Netherlands).

### 2.2. Synthesis of Mn_3_O_4_-CeO_2_ and Mn_3_O_4_-CeO_2_-rGO Nanocatalysts

For the synthesis of Mn_3_O_4_-CeO_2_, 1.2 g of cerium nitrate, 2.5 g of potassium permanganate, and 4 g of urea were completely dissolved in 30 mL of deionized water for 30 min. After that, the solution entered into the autoclave and was placed in the furnace for 14 h at a temperature of 120 °C. The resulting materials were washed several times with deionized water and dried at a temperature of 40 °C and then calcinated at 350 °C.

The hybrid of Mn_3_O_4_-CeO_2_ with reduced graphene oxide was prepared with the same synthesis used for Mn_3_O_4_-CeO_2_, except that 50 mg GO, synthesized by the Hummers method [45], was added to the solution.

Graphene oxide (GO) was synthesized by the Hummers method. In this regard, 1 g graphite powder was dispersed in 25 mL H_2_SO_4_, and then 1 g sodium nitrate was dissolved in the solution and stirred for 1 h. After that, the beaker was placed in an ice-water bath, and 3.6 g of potassium permanganate was added to the solution and stirred for 2 h. The temperature was increased to 35 °C and stirred for another 2 h, and 23 mL of deionized water was added to the above mixture and stirred for 30 min at 90 °C. Finally, the reaction was stopped by adding 70 mL of deionized water and 8 mL of H_2_O_2_ solution (30%). The product was washed with HCl (3%) and deionized water three times and dried in an oven at 50 °C.

### 2.3. Electrochemical Studies

Electrochemical studies were performed with a three-electrode system including Ag/AgCl, platinum wire, and working electrodes. The catalyst-modified glass carbon electrode (GCE) was used as a working electrode. To modify the GCE, a slurry containing 5 mg of each of the catalysts was prepared in 1 mL of a deionized water/isopropyl alcohol solution. A certain amount of slurry was placed on the GCE surface. The electrode was dried at 20 °C for 20 min and prepared for electrochemical tests.

## 3. Results and Discussion

### 3.1. Characterization

**Figure 1:**
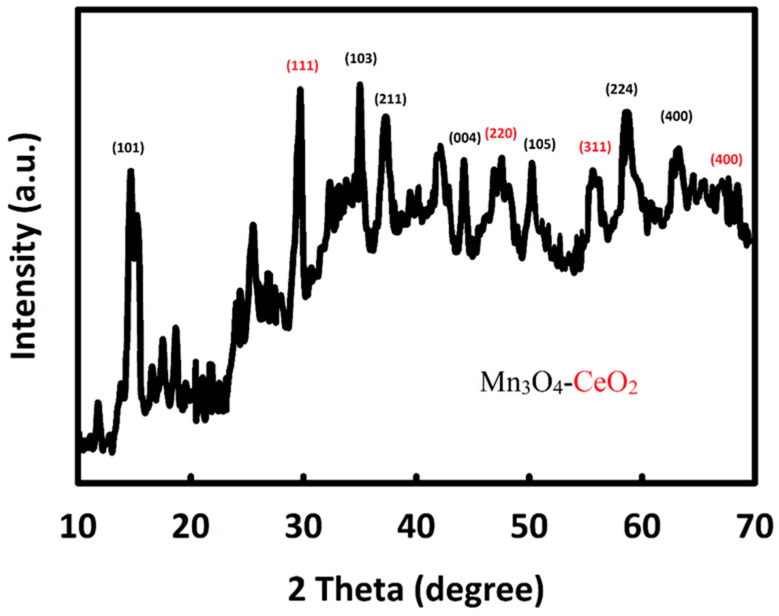
The XRD pattern of Mn_3_O_4_-CeO_2_-rGO.

The crystal structure of Mn_3_O_4_-CeO_2_-rGO nanocatalyst was examined by XRD analysis. As shown in Figure 1, the characteristic peaks of CeO_2_ were seen at the diffraction angles of about 28.9°, 47.9°, 56.8°, and 69.7°, which belonged to (111), (220), (311), and (400), crystal planes, respectively, which is in full compliance with JCPDS card no 34-0394 [46]. In addition, at diffraction angles of 17.9°, 38.1°, 36.1°, 44.4°, 50.1°, 59.8°, and 64.6°, characteristic peaks of Mn_3_O_4_ could be seen, which were related to the (101), (103), (211), (004), (105), (224), and (400) crystal planes, respectively. This diffraction pattern is in full compliance with JCPDS card no 24-0734 [47]. From XRD results, the synthesis of Mn_3_O_4_-CeO_2_-rGO was confirmed.

**Figure 2:**
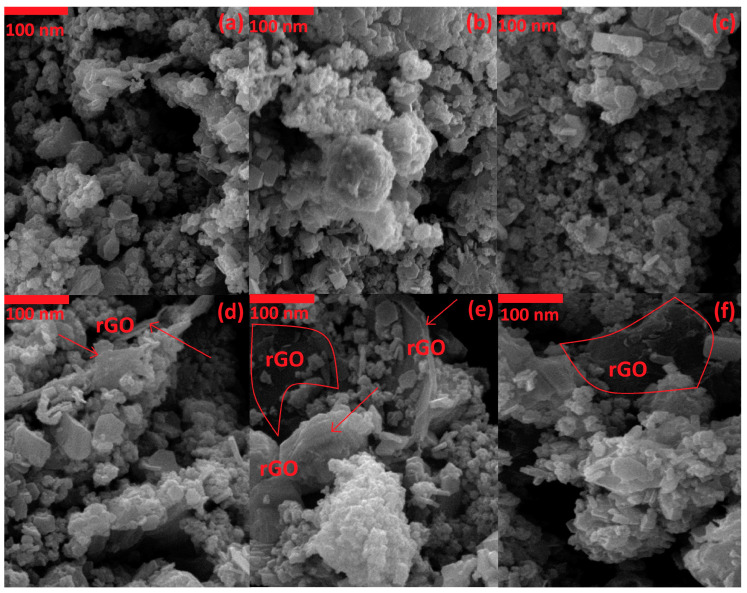
The SEM images of Mn_3_O_4_-CeO_2_ (**a**–**c**) and Mn_3_O_4_-CeO_2_-rGO (**d**–**f**).

The surface morphology of the catalysts was examined by SEM images. Figure 2a–c refer to the Mn_3_O_4_-CeO_2_ and show the porous morphology and shortcuts that are useful for methanol to reach the depth of the catalyst. In the SEM images of Mn_3_O_4_-CeO_2_ (a–c), which were prepared at the scale of 100 nm, uniform dispersion and interconnectedness of Mn_3_O_4_ and CeO_2_ can be seen. In these images, holes are seen that are shortcuts for electrolyte and methanol to penetrate the core of the catalyst. The presence of porosity and the same shortcuts facilitate the process of methanol oxidation. The SEM images of Mn_3_O_4_-CeO_2_-rGO (Figure 2d–f) show the uniform dispersion of Mn_3_O_4_-CeO_2_ on the surface of rGO nanosheets. Mn_3_O_4_-CeO_2_ covered almost the entire surface of rGO, and rGO plates were visible in some places, as shown in Figure 2d–f. In these pictures, these nanosheets are shown with an arrow or a red line around the rGO sheets.

### 3.2. Electro-Catalytic Investigations

**Figure 3:**
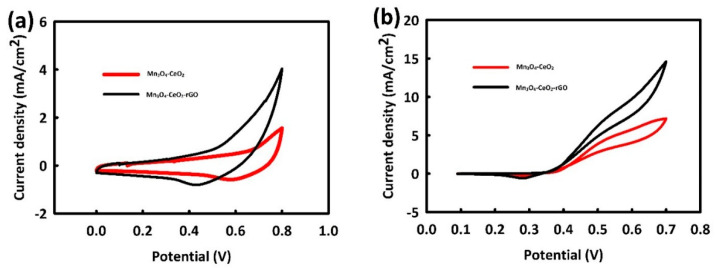
CV curves of Mn_3_O_4_-CeO_2_ and Mn_3_O_4_-CeO_2_-rGO in the absence (**a**) and in the presence of 0.2 M methanol and (**b**) in 1 M KOH.

The capability of Mn_3_O_4_-CeO_2_ and Mn_3_O_4_-CeO_2_-rGO catalysts was evaluated in the methanol oxidation process by performing cyclic voltammetry (CV) in 1 M KOH in the presence and absence of methanol. For this purpose, CV analysis of Mn_3_O_4_-CeO_2_ and Mn_3_O_4_-CeO_2_-rGO was performed in 1 M KOH solution in the potential range of 0 to 0.8 V at a scan rate of 10 mV/s. As shown in Figure 3a, both catalysts had capacitive behavior, and the current density for the Mn_3_O_4_-CeO_2_-rGO catalyst was significantly higher than that of Mn_3_O_4_-CeO_2_ due to the presence of rGO in its structure. By adding methanol (0.2 M) to the 1 M KOH solution and performing a CV test, we could see methanol oxidation peaks in both catalysts (Figure 3b), which is evidence of the relatively good electrocatalytic activity of both catalysts in the MOR process. The comparison of the behavior of two catalysts in MOR indicated the effective role of rGO in the structure of Mn_3_O_4_-CeO_2_-rGO. In addition to increasing the electrical conductivity of the catalyst, rGO increased the active surface area of the catalyst [48], and, as a result, the current density increased, and the overvoltage reduced in the methanol oxidation reaction (MOR) process. Methanol oxidation peaks for Mn_3_O_4_-CeO_2_-rGO and Mn_3_O_4_-CeO_2_ were seen at 0.51 and 0.53 V, respectively.

**Figure 4:**
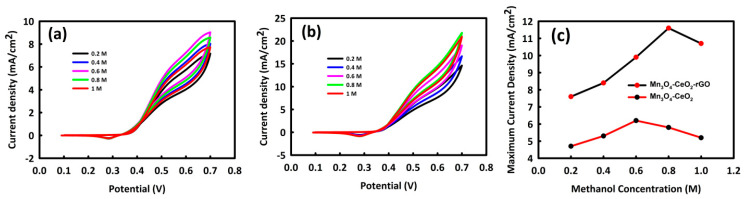
CV curves of Mn_3_O_4_-CeO_2_ (**a**) and Mn_3_O_4_-CeO_2_-rGO (**b**) at different concentrations of methanol, and (**c**) methanol concentration in terms of maximum current density for Mn_3_O_4_-CeO_2_-rGO and Mn_3_O_4_-CeO_2_.

To obtain the optimal concentration of methanol in the MOR process, CV analysis of Mn_3_O_4_-CeO_2_ and Mn_3_O_4_-CeO_2_-rGO was performed at different concentrations of methanol (0.2, 0.4, 0.6, 0.8, and 1 M) and 1 M KOH. CV analysis of the GCE modified with Mn_3_O_4_-CeO_2_ (Figure 4a) showed an upward trend of methanol oxidation peak to a concentration of 0.6 M, and from this concentration onwards, we could see a decrease in oxidation peak. For Mn_3_O_4_-CeO_2_-rGO, the oxidation peak trend was ascending to a concentration of 0.8 M (Figure 4b), and at a concentration of 1 M methanol, the current density decreased. The reason for the decrease in current density from an optimal concentration onwards is probably due to the saturation of the catalyst surface by the by-products of methanol oxidation. The likely reason is that the catalysts containing rGO are saturated later; the more effective surface of this catalyst is due to the presence of rGO in its structure. Figure 4c shows a plot of methanol concentration in terms of maximum current density. The behavior of nanocatalysts at different concentrations can be compared according to this plot.

**Figure 5:**
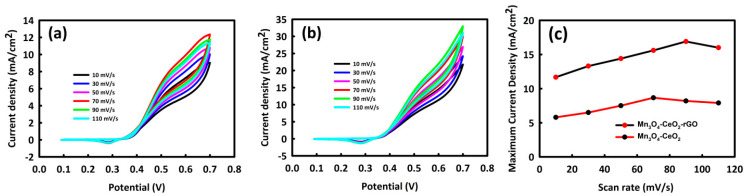
CV curves of Mn_3_O_4_-CeO_2_ (**a**) and Mn_3_O_4_-CeO_2_-rGO (**b**) at different scan rates and (**c**) different scan rates in terms of maximum current density for the Mn_3_O_4_-CeO_2_-rGO and Mn_3_O_4_-CeO_2_.

By selecting the optimal concentrations of methanol for Mn_3_O_4_-CeO_2_ and Mn_3_O_4_-CeO_2_-rGO, which are 0.6 and 0.8 M, respectively, we then investigated the behavior of these catalysts at different scan rates in the presence of a 1 M KOH solution as an electrolyte (Figure 5a,b). It was observed that with increasing scan rate, the oxidation peak current density for Mn_3_O_4_-CeO_2_ had an increasing trend until the scan rate of 70 mV/s, and from this scan rate onwards, the current density decreased. For Mn_3_O_4_-CeO_2_-rGO, it was also observed that the anodic peak current density increased up to a scan rate of 90 mV/s, and after that, the current density decreased. It is likely that at higher scan rates, the electrolyte and methanol do not have enough time to fully engage with the catalyst and penetrate to its core, reducing current density [49]. The current density for Mn_3_O_4_-CeO_2_ at scan rates of 10, 70, and 110 mV/s were 5.9, 8.8, and 7.9 mA/cm^2^, respectively, and for Mn_3_O_4_-CeO_2_-rGO at scan rates of 10, 90, and 110 mV/s were 11.6, 17.7, and 16.05 mA/cm^2^ (the selected scan rates are the lowest, optimal, and the maximum scan rates, respectively). The graph of catalyst behavior in different scan rates in terms of maximum current density is plotted in Figure 5c.

The proposed mechanism of methanol oxidation by the catalyst is as follows (Equations (1)–(4)):

(1) $${\mathrm{Mn}}_{3}O_{4}-{\mathrm{CeO}}_{2}-\mathrm{rGO}\;+{\mathrm{CH}}_{3}\mathrm{OH}\to {\mathrm{Mn}}_{3}O_{4}-{\mathrm{CeO}}_{2}-\mathrm{rGO}\;-{\mathrm{CH}}_{3}{\mathrm{OH}}_{\mathrm{ads}}$$

(2) $${\mathrm{Mn}}_{3}O_{4}-{\mathrm{CeO}}_{2}-\mathrm{rGO}\;-{\mathrm{CH}}_{3}{\mathrm{OH}}_{\mathrm{ads}}+4{\mathrm{OH}}^{-}\to {\mathrm{Mn}}_{3}O_{4}-{\mathrm{CeO}}_{2}-\mathrm{rGO}\;-{\left(\mathrm{CO}\right)}_{\mathrm{ads}}+4H_{2}O+4e^{-}$$

(3) $${\mathrm{Mn}}_{3}O_{4}-{\mathrm{CeO}}_{2}-\mathrm{rGO}\;+{\mathrm{OH}}^{-}\to {\mathrm{Mn}}_{3}O_{4}-{\mathrm{CeO}}_{2}-\mathrm{rGO}\;-{\mathrm{OH}}_{\mathrm{ads}}+e^{-}$$

(4) $${\;\mathrm{Mn}}_{3}O_{4}-{\mathrm{CeO}}_{2}-\mathrm{rGO}-{\mathrm{CO}}_{\mathrm{ads}}+{\mathrm{Mn}}_{3}O_{4}-{\mathrm{CeO}}_{2}-\mathrm{rGO}\;-{\mathrm{OH}}_{\mathrm{ads}\;}+{\mathrm{OH}}^{-}\to {\mathrm{Mn}}_{3}O_{4}-{\mathrm{CeO}}_{2}-\mathrm{rGO}+{\mathrm{CO}}_{2}+H_{2}O+e^{-}$$

The mechanism of methanol oxidation in alkaline media at the surface of these catalysts can be divided into three stages, including adsorption of methanol, adsorption of hydroxyl ions, and breaking the C–H and O–H bonds of methanol to produce products such as CH_2_OH, CHOH, COH, and CO [50]. In the adsorption process, likely the synergetic effect of Mn_3_O_4_, CeO_2_, and rGO improves this process by creating more active sites. Hydroxyl ions adsorbed on the surface of the catalyst also help to oxidize the adsorbed CO, thereby regenerating the active sites of the catalyst. Since the active sites of catalysts are important in the MOR process, with the addition of rGO, the overall performance of the catalyst increases.

**Figure 6:**
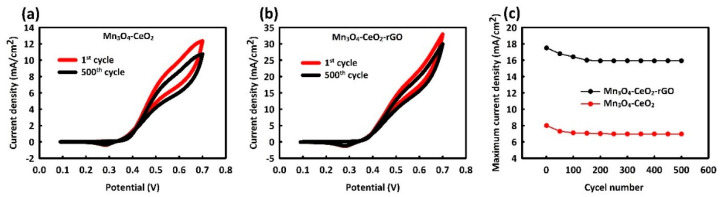
The first and 500th CV cycles of Mn_3_O_4_-CeO_2_ (**a**) and Mn_3_O_4_-CeO_2_-rGO (**b**) and plot of maximum current density versus cycle number (**c**).

To evaluate the stability of Mn_3_O_4_-CeO_2_ and Mn_3_O_4_-CeO_2_-rGO catalysts, 500 CV cycles were performed at optimal methanol concentrations and scan rates (0.6 M in scan rate of 70 mV/s for Mn_3_O_4_-CeO_2_ and 0.8 M in scan rate of 90 mV/s for Mn_3_O_4_-CeO_2_-rGO). As seen in Figure 6a, Mn_3_O_4_-CeO_2_ showed stability of about 87%, while the stability achieved 91% for Mn_3_O_4_-CeO_2_-rGO (Figure 6b). Examination of the current density against the number of cycles is shown in Figure 6c, indicating Mn_3_O_4_-CeO_2_ reached relatively good stability from about 200 cycles onwards, and Mn_3_O_4_-CeO_2_-rGO achieved stability in current density approximately in the 250th cycle. The greatest reduction in current density in the initial cycles for both Mn_3_O_4_-CeO_2_ and Mn_3_O_4_-CeO_2_-rGO catalysts was seen. It seems that in the initial cycles, methanol and electrolyte did not find enough time to penetrate the catalyst nucleus, and over time, and in subsequent cycles, methanol and catalyst came into full contact with each other, and after a slight decrease, stability in the current density was observed [51]. Moreover, the CV shape of catalysts remained stable after different cycles, and no change was seen in isopotential points, which is the reason for the very good structural stability of the catalyst [52].

**Figure 7:**
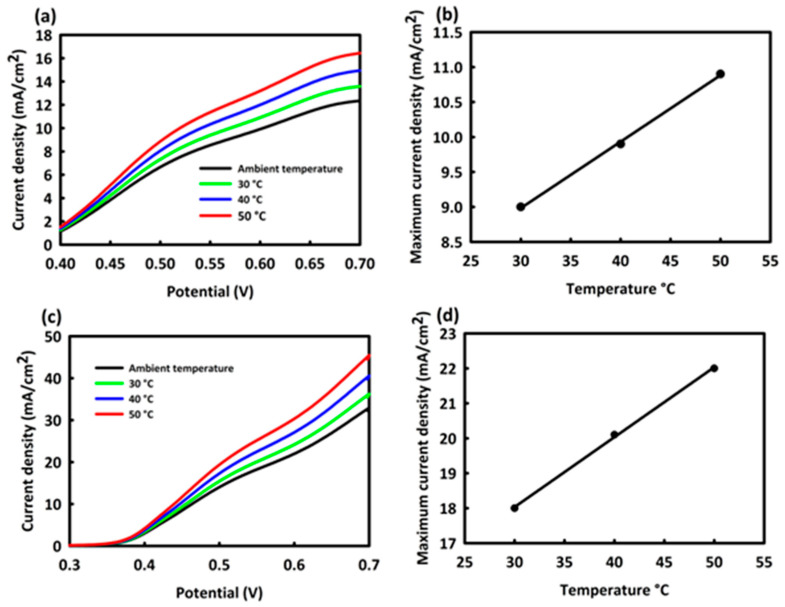
CV curves at different temperatures and plots of maximum current density against temperature (at 30–50 °C) for Mn_3_O_4_-CeO_2_ (**a**,**c**) and Mn_3_O_4_-CeO_2_-rGO (**b**,**d**).

The effect of temperature in the MOR process on Mn_3_O_4_-CeO_2_ and Mn_3_O_4_-CeO_2_-rGO catalysts was evaluated by performing a linear sweep voltammetry LSV test at an optimal concentration of methanol and scan rate. As seen in Figure 7a,c, the current density in both catalysts increased with increasing temperature, indicating that the increasing temperature facilitates the process of methanol adsorption on the surface of the catalyst. The rate of increase of current density with growing temperature for Mn_3_O_4_-CeO_2_-rGO was slightly higher than Mn_3_O_4_-CeO_2_; here too, it is likely that rGO played an effective role in increasing the active surface area of the catalyst. The two parameters of maximum current density and temperature were linearly related to each other, which can be seen for Mn_3_O_4_-CeO_2_ and Mn_3_O_4_-CeO_2_-rGO in Figure 7b,d, respectively.

## 4. Conclusions

In the energy-dependent modern world, we see every day the introduction of new and high-efficiency materials for use in the structure of energy storage and production devices. In this regard, we synthesized a stable and inexpensive nanocatalyst based on metal oxides (Mn_3_O_4_-CeO_2_-rGO) with a one-step and easy synthesis hydrothermal method for use in the methanol oxidation process. The structure and morphology of nanocatalyst were evaluated by XRD and SEM. In this study, the synergetic effect and effective role of rGO in the catalyst structure were investigated. Mn_3_O_4_-CeO_2_-rGO with 91% cyclic stability after 500 consecutive CV cycles and a maximum current density of 17.7 mA/cm^2^ at an overvoltage of 0.51 V (at scan rate and optimum concentration) can be an attractive and new option for use as a catalyst for methanol oxidation.

## Data Availability

The data presented in this study are available upon request from the corresponding authors.
